# Additional factors to corelate with a more than 30% NIHSS score improvement in patients 7 days after fibrinolytic and/or endovascular treatment for ischemic stroke

**DOI:** 10.1186/s12883-020-01990-z

**Published:** 2020-11-14

**Authors:** Raluca Tudor, Gheorghe Iovanescu, Daniela Reisz, Amalia Cornea, Cristina Potre-Oncu, Adrian Tutelca, Mihaela Simu

**Affiliations:** 1 Department of Neurology, University of Medicine and Pharmacy “Victor Babes”, Timisoara, Romania; 2 Department of Otorhinolaryngology, University of Medicine and Pharmacy “Victor Babes”, Timisoara, Romania; 3 Department of Hematology, University of Medicine and Pharmacy “Victor Babes”, Timisoara, Romania; 4 Department of Interventional Radiology, Timis County Emergency Clinical Hospital “Pius Branzeu”, Timisoara, Romania

**Keywords:** Diabetes mellitus, NIHSS, ASPECTS, Hyperglycemia, Thrombolytic treatment

## Abstract

**Background:**

Our objective was to find which additional factors can influence the favorable result in stroke patients after receiving fibrinolytic and/or endovascular treatment, quantified as a more than 30% improvement of the NIHSS score at 7 days.

**Methods:**

This is a retrospective study to find factors that could influence a favorable evolution of patients with stroke that underwent fibrinolytic and or thrombectomy using the NIHSS score changes. At the admission in the hospital, blood glucose, blood count, coagulation time, INR, aPTT, PT, platelet count, NIHSS questionnaire and ASPECTS score were collected. NIHSS was assessed at the admission, after 1 h, after 2 h, after 24 h and after 7 days.

**Results:**

As compared to the initial evaluation, at 7 days after admission 59% (72) of patients have improved with more than 30% the NIHSS. Higher levels of systolic blood pressure, glycemia and lower ASPECTS score at admission were observed in non-achievers. The value of INR contributed to model: for every unit increase of INR, the chance of better outcome decreases by 90,1%. High glycemia has also a negative impact: for every unit increase, the chance of better outcome decreases by 24%. Higher initial ASPECTS score is associated with better outcomes: each point increase of ASPECTS score at initial evaluation, increases the chance of better outcome by 154.2%.

**Conclusion:**

Males, older age, diabetes, and hyperglycemia correlate with a worse outcome after cerebral stroke regardless of the benefit yielded fibrinolytic and/or thrombectomy therapy. In this study, patients with the above-mentioned factors did not improve more than 30% of baseline NIHSS score from admission to the 7th day.

## Background

Due to its major socio-economic burden, stroke represents a significant cause of mortality and disability worldwide. Therefore, there is a permanent need to enhance our knowledge on its prognosis and comorbidities [1, 2].

Stroke is a complex pathology, and its identification and diagnosis is initially based on the clinical examination of the patient. Intravenous thrombolysis and mechanical thrombectomy are efficacious and safe in patients with acute ischemic stroke, and earlier treatment is associated with better outcome [3].

One of the major risk factors for stroke is represented by diabetes which accounts for approximately 3–20% of stroke risk [4]. Compared with non-diabetic patients, the risk of stroke is 2–6 times higher in patients with diabetes [5]. Although these findings have been well documented, the link between admission glucose level and stroke outcome is still a field for ongoing research.

Moreover, hyperglycemia is also associated with poor prognosis even in non-diabetic patients, both in terms of functional recovery, and mortality, regardless of the patient’ age, stroke sub-type and severity [6].

Other predictors of outcome are age, gender, previous smoking status and treatment with statines, atrial fibrillation, baseline National Institutes of Health Stroke Scale (NIHSS) score, CT findings, and time to treatment and recanalization [7].

,An extensively used scale for evaluating neurological function in stroke patients, that can reflect the patients neurological deficits and accurately determine the prognosis, is NIHSS [8]. The NIHSS score was initially created as a research tool to evaluate baseline data on patients with acute stroke. Due to the fact that it is a good predictor of both short- and long-term outcome of stroke patients it is now used as a clinical evaluation tool to neurologically assess stroke patients, to determine both an better treatment and predict the patients’ outcome [9].

The NIHSS is composed of a 15-item neurologic examination stroke scale that examines impairment in 11 domains and evaluates the effect of acute cerebral infarction on the levels of: consciousness, language, neglect, visual-field loss, extraocular movement, motor strength, ataxia, dysarthria, and sensory loss. Each item is scored with 3 to 5 grades, zero representing a normal function. A higher score represents an increased deficiency. The scores from each item are summed in a total score. The greater the score the higher the deficit and stroke severity [9].

The current imagistic standard for patients with acute stroke in the first 3 h after the onset of symptoms is computed tomography (CT). this analysis is sufficient not only exclude intracerebral haemorrhage but also to exclude early signs of infarction [10–12].

The Alberta Stroke Program Early CT score (ASPECTS) is well grounded tool that detects early ischemic changes on non-contrast CT scans of the brain, in patients that are suspected of having anterior circulation occlusion. It is used as a screening tool for acceptability in receiving interventional mechanical thrombectomy treatment [13].

ASPECTS consists of a 10-point quantitative score. For any evidence of early ischemic change for each of the defined region 1 point is subtracted from 10. Score of 0 indicates infarction of all 10 regions [13].

Early evaluation of results, after 7 or 30 days, could enhance the efficacy of exploratory trials and minimize biases from unrelated adverse events. A study that analyzed 10,000 random samples of patients with diabetes, divided into treated and untreated groups, found that the NIHSS score at day 7 appears to be a sensitive end point that should be validated in randomized trial datasets to be used in exploratory stroke trials [9].

Several studies have established that the baseline National Institutes of Health Stroke Scale (B-NIHSS) is a predictor of functional outcome [14, 15], but only few studies are focused on the functional relevance of the NIHSS evolution in the first 24 h and 7-day after stroke onset [16–18].

Our objective was to find additional factors that might influence the favorable evolution of patients with stroke after receiving fibrinolytic and/or endovascular treatment, by quantifying it as a more than a 30% improvement of NIHSS at day 7.

## Methods

This is a retrospective study to find factors that could influence a favorable evolution of patients with stroke using the NIHSS score. The study was performed at the Timisoara County Emergency Clinical Hospital during 2017–2019. During this period, 329 patients who were diagnosed with acute stroke, were admitted in Neurology department. We included in this study all the patients who met the national and international criteria for fibrinolytic and/or endovascular treatment of acute stroke. Inclusion criteria for the study were: age over 18 years, patients with signs and symptoms of acute stroke, brain hemorrhage excluded by computed tomography, symptom onset ≤4 h and 30 min until the initiation of bolus i.v, BP < 185/110 before initiation and during thrombolysis, blood glucose > 50 mg/dl Exclusion criteria were those generally used according to the national protocol for thrombolysis: history of cerebral hemorrhage, history of known ischemic stroke in the last 3 months or neurosurgical procedures (intracranial/spinal) in the previous 3 months, intra-axial brain tumor). Exclusion criteria related to coagulopathies: INR > 1.71, aPTT> 40 s, PT > 15 s, platelet< 100.000/mmc^2^, treatment with oral non-antivitamin K oral anticoagulants in the last 48 h, treatment with unfractionated heparin with aPTT>40s and treatment with low molecular weight heparin at therapeutic dose during the last 24 h.

After we applied the inclusion and exclusion criteria, 122 patients were included in the study. From the 122 patients, 108 received thrombolytic treatment and 14 received endovascular treatment through mechanic thrombectomy. mTICI post-thrombectomy score was 3 for 9 patients, 2b for 2 patients, 2a for 1 patient and 0 for 2 patients. Time from debut until treatment initiation for the 122 included patients was between 40 and 346 min. Unfortunately, we do not have data for the pre-stroke MRS score which was analyzed only post-stroke. We excluded 202 patients who did not met the criteria for thrombolysis and/or thrombectomy and 5 patients for incomplete data at 7 days evaluation. For this study, each patient provided the informed consent to use the medical data. The study was approved by the Ethics Committee of the hospital nr.190/05/05/2020.

At hospital admission, blood glucose, blood count, coagulation time, INR, aPTT, PT, platelet count, were collected and NIHSS and ASPECTS scores were applied. NIHSS was assessed at the admission (baseline), 1 h, 2 h, 24 h after the procedure and then 7 days later.

### Statistical analysis

Continuous data are presented as mean +/− standard deviation or median (inter-quartile range) and categorical data are presented as percentages. For comparison of proportions chi-square test or Fisher Exact tests were used, as appropriate. With a 2-category factor, for parametric data t-test was used, and for non-parametric or ordinal data, the Mann-Whitney test was used. The evolution of NIHSS Score was tested with a general linear model for repeated design. A logistic regression model was built, with achievement of at least 30% of improvement of NIHSS between initial evaluation and evaluation at 7 days as a dependent variable, and demographic and clinical factors as independent variables.

## Results

In Table 1 are presented demographic and clinical characteristics of patients included in the study at admission.

**Table 1:** Demographic and clinical characteristics of patients

	*N* = 122
Male gender, *n (%)*	75 (61.5%)
Prior diagnostic of hypertension, *n (%)*	86 (70.5%)
Prior diagnostic of stroke, *n (%)*	55 (45.1%)
Prior diagnostic of diabetes mellitus, *n (%)*	13 (10.7%)
Prior diagnostic of transient ischemic attack, *n (%)*	5 (4.1%)
Dyslipidemia, *n (%)*	57 (46.7%)
Atrial fibrillation, *n (%)*	39 (32.0%)
Prior diagnostic of myocadiac infarction *n (%)*	9 (7.4%)
Prior diagnostic of valvopathies, *n (%)*	3 (2.5%)
Prior anticoagulant treatment, *n (%)*	6 (4.9%)
Prior antiplatelet therapy, *n (%)*	38 (31.1%)
Age, *mean (SD)*	67.3 (12.0
Systolic BP at admission, *mean (SD)*	155.3 (21.8
Diastolic BP at admission, *mean (SD)*	80.4 (12.9
No of platelets (thousands), *mean (SD)*	213.8 (60.2
Hemoglobin, *mean (SD)*	13.7 (1.6
Total cholesterol, *mean (SD)*	188.0 (45.9
ASPECTS at admission, *mean (SD)*	9.5 (0.9
INR, *median (IQR)*	1.015 (0.13)
Glycemia, *median (IQR)*	129.0 (61)
Creatinine, *median (IQR)*	0.9 (0.27)
Triglycerides, *median (IQR)*	104.0 (69)

**Fig. 1:**
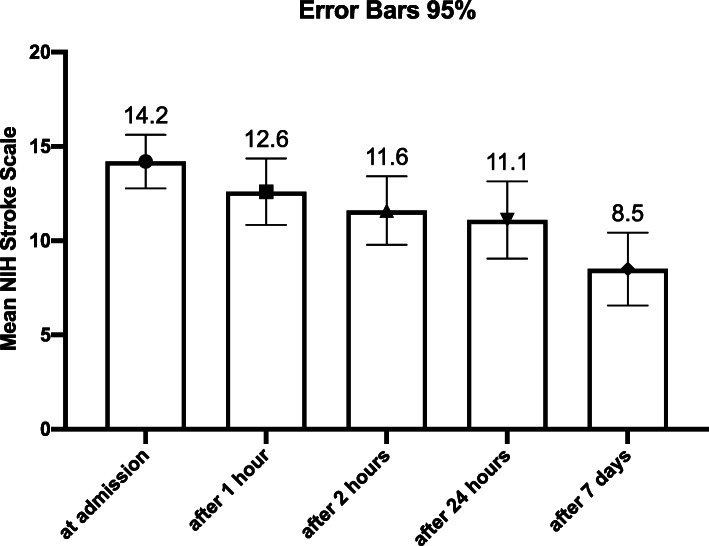
Evolution of NIHSS from admission to the evaluation at 7 days

In order to assess the evolution of NIHSS we applied a general linear model for repeated design and found a linear significant decrease between each step of the evaluation, F(4,108) = 25.8, *p* < 0.001, with a mean decrease between the evaluation at admission and after 7 days of 5.438 with a 95% CI between (3.894; 6.981) (Fig. 1).

As compared to the initial evaluation, at 7 days after admission, 72 (59%) of patients have improved the NIHSS score with more than 30%. Furthermore, all the demographical and clinical characteristics of patients were compared between the group that had succeeded to increase the initial score more than 30%, and those that did not (Table 2). Significant higher proportions of men, patients diagnosed with diabetes mellitus were found in the group that did not succeed to increase with at least 30% the NIHSS at 7 days. Also, higher levels of systolic blood pressure, glycemia and lower ASPECTS score at admission were observed in non-achievers.

**Table 2:** Comparison of demographic and clinical by improvement of NIHSS with more than 30% at 7 days

Improvement of NIH stroke scale with more than 30% at 7 days			
	No	Yes	*p*-values
Time to initiation of treatment (min), *mean (SD)*	172.7 (45.7)	157.7 (47.7)	0.079 ^b^
Gender male, *n (%)*	38 (76.0%)	37 (51.4%)	0.006^a^
Prior diagnostic of hypertension, *n (%)*	33 (66.0%)	53 (73.6%)	0.365^a^
Prior diagnostic of stroke, *n (%)*	30 (60.0%)	25 (34.7%)	0.006^a^
Prior diagnostic of diabetes mellitus, *n (%)*	3 (6.0%)	10 (13.9%)	0.165^a^
Prior diagnostic of transient ischemic attack, *n (%)*	3 (6.0%)	2 (2.8%)	0.399^a^
Dyslipidemia, *n (%)*	25 (50.0%)	32 (44.4%)	0.545^a^
Atrial fibrillation, *n (%)*	15 (30.0%)	24 (33.3%)	0.689^a^
Prior diagnostic of myocadiac infarction, *n (%)*	2 (4.0%)	7 (9.7%)	0.306^a^
Prior diagnostic of valvopathies, *n (%)*	1 (2.0%)	2 (2.8%)	0.999^a^
Prior anticoagulant treatment, *n (%)*	3 (6.0%)	3 (4.2%)	0.688^a^
Prior antiplatelet therapy, *n (%)*	13 (26.0%)	25 (34.7%)	0.306^a^
Age, *mean (SD)*	67.5 (12.4)	67.2 (11.9)	0.890^b^
Systolic BP at admission, *mean (SD)*	160.2 (21.1)	151.9 (21.7)	0.037^b^
Diastolic BP at admission, *mean (SD)*	81.1 (13.7)	79.9 (12.3)	0.615^b^
No of platelets (thousands, *mean (SD)*	207.2 (63.8)	218.4 (57.7)	0.312^b^
Hemoglobin, *mean (SD)*	14.1 (1.6)	13.5 (1.6)	0.055^b^
Total cholesterol, *mean (SD)*	191.2 (46.9)	185.8 (45.5)	0.539^b^
ASPECTS at admission, *mean (SD)*	9.2 (1.0)	9.7 (0.7)	0.008^b^
INR, *median (IQR)*	1.0 (0.15)	1.0 (0.13)	0.138^c^
Glycemia, *median (IQR)*	153.5 (85)	119.0 (42)	< 0.001^c^
Creatinine, *median (IQR)*	0.9 (0.3)	0.9 (0.24)	0.313^c^
Triglycerides, *median (IQR)*	102.5 (67)	105.0 (66)	0.855^c^

^a^ chi-square test or Fisher Exact test, as appropriate

^b^independent samples t-test

^c^Mann—Whitney test

In order to predict which of the clinical characteristics at admission influenced the achievement of more than 30% improvement of NIHSS at day 7, we used a logistic model with the clinical characteristics at admission as covariates, with the forward conditional method. Model explains between 34.2–46.3% of the variation of dependent variable. In Table 3 are presented the beta values, the OR and the 95% CI for the OR for the independent variables with significant contribution to the model. The achievers are 3.6 more likely to be women.

**Table 3:** Prediction of the achievement of more than 30% improvement of NIHSS from admission until the 7th day from demographic and clinical factors

				95% C.I. for OR	
	B	S.E.	OR	Lower	Upper
Female gender	1.288	.536	3.627	1.270	10.361
No of platelets (thousands)	.008	.004	1.008	1.000	1.017
INR	−3.972	1.809	.019	.001	.652
Glycemia	−.024	.006	.976	.965	.987
ASPECTS at admission	.933	.305	2.542	1.397	4.622

Logistic regression with dependent variable: achievement of more than 30% improvement of NIHSS from admission until the 7th day; chi-square (5)=48.2, *p* < 0.001

The number of platelets proved a significant contribution for better outcome; for every thousand (1000) increase in number of platelets the probability of better response in 7 day increased by 0.8%. Number of platelets contribute significant to the prediction of better outcome, for every thousands of platelets the probability of better response in 7 day increases by 0.8%. For every unit increase of INR value, the chance of better outcome decreased by 90,1%. High glycemia had also a negative impact: for every unit increase, the chance of better outcome decreased by 24%. A higher initial ASPECTS score is associated with better outcomes: for every point increase at ASPECTS, the chance of better outcome increases by 154.2% (Table 3).

## Discussion

Clinical outcome after stroke is variable and depends on many factors. Due to the development of medical science and technology, the objective assessment of disease severity of patients with stroke can provide basis for prediction of prognosis and medical decisions [19].

The objective of this study was to find factors that influence the favourable evolution of patients with stroke after receiving fibrinolytic and/or endovascular treatment. The favourable evolution was quantified by improving by more than 30% of NIHSS score in 7 days. The statistical analysis revealed that platelet count could be a predictor of patients’ evolution in the first 7 days. The correlation between platelet count and NIHSS scale values has not been much studied.

We observed that when comparing to the admission evaluation, after treatment initiation and at 7 days after admission, 59% of the patients have improved with more than 30% NIHSS. For our sample the initial mean NIHSS score was 13.9 and after 7 days it reduced to 8.5. Our findings are consistent with those of Atchin and his colleagues, who analyzed the importance of baseline NIHSS score in predicting outcomes at 7 days after stroke [20].

We tested as factors of worse outcome (according to our study design, patients that did not improve more than 30% NIHSS score) gender, age, INR and glycemia at admission and ASPECTS score. We have observed that independent variables that lead to a better outcome were female and reduced number of platelets, when controlling for gender, age, glycemia and ASPECTS at admission.

Previous research have shown that hyperglycemia and diabetes mellitus are linked with microvasculature impairments, blood-brain barrier, and increased hemorrhagic infarct conversion after reperfusion [21, 22]. Diabetes mellitus and hyperglycemia are strongly linked to increased risk of symptomatic intracranial hemorrhage after thrombolytic treatment [23].

A study that included 54 reports that evaluated the effect of diabetes mellitus or admission glucose level on outcomes after thrombolysis concluded that history of diabetes mellitus and glucose level are associated with poor clinical outcome after thrombolysis [24]. Another recent article found that age of > 70 years and the presence of diabetes mellitus are significant predictors of unfavourable outcomes. Moreover, higher baseline NIHSS score and lower ASPECTS score were also independent predictors of unfavourable outcomes [25]. In our model, age and glycemia at admission did not significantly contribute to the outcome, but they were controlled for.

A study that used the electronic ASPECTS score to evaluate a large database of thrombolysis patients with acute ischemic stroke found that decreasing ASPECTS score were significantly correlated with increasing baseline NIHSS scores. Furthermore, in a univariate analysis, lower ASPECTS score was significantly associated with worse 90-day clinical outcome [26].

In a study that that included 82 patients with acute ischemic stroke treated with thrombolytic therapy, found that NIHSS score > 12 on arrival, age > 70 years and the presence of hyperdense artery sign (according to ASPECT assessment) are predictors of unfavourable outcome at 3 months [27]. According to our findings, we observed that increased initial ASPECTS score was associated with better outcomes.

Platelets play an important role in the mechanism of ischemic stroke but the effect of platelet count (PC) in the pathogenesis of stroke remains poorly understood. In a study that investigated the possibility of PC being an independent risk factor for ischemic and hemorrhagic stroke found that PC was in correlation with worse outcome for patients with hemorrhagic stroke. No correlations were found between PC outcome for patients with ischemic stroke [28]. Compared to this study, we found that the higher number of platelet can contribute significantly to the prediction of better outcome.

One advantage of this technique could be that it is easy to perform and does not involve special techniques or additional costs. A limitation of this study could be that it is retrospective and many patients have been excluded due to lack of data thus the group of patients included in the study was small.

## Conclusion

Identifying factors associated with better outcome can be useful for establishing interventions for stroke by early treatment of other conditions such as diabetes. Factors such as: men, age, patients diagnosed with diabetes, and hyperglycaemia have a worse outcome after cerebral stroke. In this study, patients with the above-mentioned factors did not improve more than 30% of NIHSS score from admission to the 7th day. We observed that higher number of platelet count can contribute significantly to the prediction of better outcome.

## Data Availability

The authors confirm that all data underlying the findings are fully available without restriction. Data can be obtained after submitting a request to the hospital.

## Abbreviations

NIHSS: National Institutes of Health Stroke Scale
ASPECTS: Alberta Stroke Program Early CT score
B-NIHSS: baseline National Institutes of Health Stroke Scale
aPTT: activated Partial Thromboplastin time
PC: Platelet count
